# The American Medical Almanac, for 1841

**Published:** 1841-01

**Authors:** 


THE AMERICAN ALMANAC, FOR 1841.

For this excellent little annual the profession is indebted to the enterprise of Dr. J. V. C. Smith, the spirited editor of the Boston Medical and Surgical Journal. It was prepared especially for the use of practising physicians, surgeons, students, and apothecaries, and embraces a greater amount of interesting and really useful information on subjects pertaining to the profession, than is anywhere to be found in the same compass. No physician or student should be without it. As a pocket manual it will prove a great convenience to the practitioner; and for reference, it is invaluable. The body of statistics which it presents, on almost every conceivable subject of interest to the medical man, is immense. We hope Dr. Smith will be encouraged to continue his almanac annually. We doubt whether there is a man in the country who would get up such a work with so much industry, judgment and taste.

We give a few extracts from the "Statistics of Insanity and Institutions for the Insane, in the United States," which is a subject of universal interest:

The recoveries from insanity in the New England institutions are about fifty per cent. of the whole number of patients admitted, and an average of from 80 to 90 per cent. of the recent cases of less than one year's duration. The term "recent case" is applied differently in different institutions. In the State Lunatic Hospital a case of less duration than one year is denominated a recent case. In some institutions the period is limited to six months, and in others to three months. This will make an obvious difference in the record of recoveries, as it is well known, that cases of three and six month's duration, recover in greater numbers than those that have existed nine and twelve months.

The present condition of the institutions in New England, New York, Pennsylvania and Ohio, is very good, perhaps better than most others in the world; and if in the other States they have heretofore been behind them, a spirit of improvement is awakened which will secure for these institutions the same advantages now possessed by their neighbors.

To Dr. Rush, our distinguished countryman, the new world is indebted for the application of the law of kindness in the management of the insane. Like the immortal Pinel, his efforts diffused light and knowledge among his countrymen, and the condition of the insane has been one of progressive improvements from his time to the present. He opened the prison door, unbarred the dungeon, and knocked off the shackles of the maniac with the same fearlessness and with the same benevolent spirit as incited the French philanthropist when he entered the dungeons of the Bicétre and liberated the tenants of half a century. The writings of Dr. Rush are full of instruction on the moral treatment of insanity, and his essay "On the Influence of Physical Causes upon the Moral Faculty," exhibits a knowledge of certain forms of mental disease, quite in advance of his cotemporaries in Europe."

The treatment of insanity in the institutions of New England, is based on the philosophy of Dr. Todd, and may be summed up in a few words—*The adaptation of the law of kindness to all cases—the inculcation of self-control by various means, always kept prominently in view of the patient, and the application of judicious medical remedies and regimen, to restore and preserve a healthy state of the physical system.*

It is only in the institutions assigned for the special treatment of insanity that this system can be carried out, so as to afford a reasonable hope of success. Home and friends so desirable to us in most cases of disease, are generally not well appreciated by the insane. Any attempt to control and manage them by friends, is generally met by resistance, and usually by enmity and opposition. The best advice of the physician is discarded, and no system can be pursued with sufficient regularity and perseverance to afford any great prospect of benefit.

In the moral treatment of the insane, the first object is to secure the confidence of the patient. This must be done by extending to them every indulgence and kindness compatible with their own safety and that of those around them, and who have them in charge. If furious and violent, every motive should be presented to induee them to be reasonable and quiet.

In their moments of composure they will be influenced by motives, and in their greatest violence, kindness and gentleness will often restore tranquillity, when severity will induce excessive fury, and permanent prejudice against those who control them.

If we desire the insane to act as rational beings, we must be consistent and treat them as such, encourage their self-respect, present to them motives of self control, and grant to them such liberties as they can appreciate and will not abuse. For this purpose labor and amusement are invaluable auxiliaries in the treatment of the insane. They require a safety valve to expend the excessive excitement of the system, and if it be not expended in labor or diversion, it will be exhibited in noise, violence, and mischief. Next to judicious medical treatment, employment is the best remedial means to remove insanity—it is a complete substitute for restraints, which are irksome, and which should never be applied when other means of control are available and effectual. Pursuing this course with perseverance, in a great proportion of the cases, the violent and furious will become calm, without any corporeal restraint whatever. But there are cases in which such restraints are necessary both to the welfare of the patient and those with whom he associates. When they are resorted to, they should be as easy and comfortable as possible, and should be removed as soon as the patient is in a candition to control himself. The solitary room, the mittens, and the wrist-bands, are all the corporeal restraints admitted in the State Lunatic Hospital, and while this paper is being written, with 240 patients, not exceeding five are under any such restraints whatever.

While restraints should be applied as rarely as possible, and never but for the personal benefit of the patient, and the safety of those with whom he must associate, yet they are important auxiliaries in the treatment of the insane, and I can but deprecate the plan of dispensing with them entirely, as the current of popular opinion at present seems to tend.

It is undoubtedly true, that with corporeal restraints judiciously applied, the patient will sooner be made tranquil than by the presence of one or more attendants, who he well knows watch all his movements, and will interfere with all his mischievous designs. Their presence will become a source of irritation itself, which will serve to keep up his excitement.

Restraints are no less necessary for the suicidal melancholic than for the furious maniac, and for both are far less disagreeable to the patient than the unceasing *surveillance* which is necessary as a substitute. Labor is the best substitute for restraint, as it expends to good purpose the excitement which is the cause of the noise and mischief with the violent insane. Suitable labor presents a constant motive to regularity, concentrates the mind to one object, exhausts in muscular action the accumulated excitability, promotes healty appetite and quiet sleep.

Labor is a rational employment, and the insane feel no little complacency in being able to perform it acceptably.

Disease, with the maniac, is a substitute for sleep. It keeps the brain perpetually active in the *production* of the principle of life as well as in its *expenditure.*

The object of medicinal remedies in the treatment of insanity is two fold. To remove the symptoms of bodily disease that may be present, and not unlikely the cause of the disease which affects the mind, and to act directly upon the brain and nervous system, influencing their condition, and removing all irritation which keeps up maniacal excitement.

Experience shows that many of the causes of insanity are to be found in the diseased condition of organs, connected only by sympathy with the brain. These cannot be reached by moral influence or restraints, but must be removed by the application of suitable medicinal remedies adapted to their cure. There is also a condition of the brain itself on which insanity often depends, which may be reached by these remedies with as much certainty as other diseases are relieved by medicines. The maniacal excitement subsides as the brain becomes restored to a healthy condition.

Those physicians who only prescribe moral treatment for the insane, overlook the important fact that insanity is a physical disease, always depending on a morbid condition of the brain and nerves, primarily induced, or the result of direct contact with diseased functions, in sympathy with them, with the existence of which a restoration to health and soundness is wholly incompatible. In this consists the superior advantage of the system of treatment pursued in all the New England institutions for the insane, that while they adopt the very best moral treatment, they also rely with great confidence on a careful, judicious, but efficient use of remedial agents. Combined, they render insanity a less formidable disease than many others less terrific, and enable us to restore from 80 to 90 per cent. of cases, a success that can hardly be claimed for any other formidable disease.

Rhode Island, Delaware, North Carolina, Illinois, Indiana, Mississippi, Missouri, Louisiana, Alabama, Michigan, and the Territories, with a population of 2,300,000, have no institutions for the Insane, and have made no efforts to raise the funds necessary to establish one. The number of insane in these States and Territories, is probably about 2,300.

Massachusetts, about 700 insane—accommodations for 480; Maine, 400 insane—accommodations for 120; Vermont, 250 insane—accommodations for 70; Connecticut, 300 insane—accommodations for 100; New York, 1,800 insane—accommodations completed for 430, preparing for 800—1,230; Pennsylvania, 1,400 insane—accommodations for 300; Maryland, 360 insane—accommodations for 80; Virginia, 1,000 insane—accommodations for 300; Ohio, 1,000 insane—accommodations for 120; Kentucky, 550 insane—accommodations for 130.

Dr. Woodward, the distinguished superintendent of the Worcester Lunatic Hospital, by whom the above statistical table was constructed, supposes that there is in New England, 1 insane person suitable for confinement in a hospital to 1,000 of the population. At the south the proportion is somewhat less.

The Statistics of Phrenology are given with the rest, and justice is done, in the following extract, to the great AMERICAN PHRENOLOGIST:

*Dr. Charles Caldwell.*—This gentleman has ever been a most able and efficient advocate of Phrenology. His writings in its behalf have been very numerous; if they were all collected and published together, they would constitute three or four large octavo volumes. His labors and merits will be more appreciated, as well as held in greater respect, by posterity than by the present generation.

